# Development of amine-functionalized fluorescent silica nanoparticles from coal fly ash as a sustainable source for nanofertilizer

**DOI:** 10.1038/s41598-024-53122-z

**Published:** 2024-02-06

**Authors:** Vikram Singh, Tuhin Mandal, Shiv Rag Mishra, Anupama Singh, Puja Khare

**Affiliations:** 1 Environment Emission and CRM Division, CSIR-Central Institute of Mining and Fuel Research Dhanbad, Dhanbad, Jharkhand 828108 India; 2 Coal to Hydrogen Energy for Sustainable Solutions, CSIR-Central Institute of Mining and Fuel Research Dhanbad, Dhanbad, Jharkhand 828108 India; 3 Academy of Scientific and Innovative Research (AcSIR), Ghaziabad, 201002 India; 4 Agronomy and Soil Science Division, CSIR-Central Institute of Medicinal and Aromatic Plants, Lucknow, Uttar Pradesh 226015 India

**Keywords:** Plant sciences, Environmental sciences, Materials science, Nanoscience and technology

## Abstract

Scaling up the synthesis of fluorescent silica nanoparticles to meet the current demand in diverse applications involves technological limitations. The present study relates to the hydrothermal synthesis of water-soluble, crystalline, blue-emitting amine-functionalized silica nanoparticles from coal fly ash sustainably and economically. This study used tertiary amine (trimethylamine) to prepare amine-functionalized fluorescent silica nanoparticles, enhancing fluorescence quantum yield and nitrogen content for nanofertilizer application. The TEM and FESEM studies show that the silica nanoparticles have a spherical morphology with an average diameter of 4.0 nm. The x-ray photoelectron and Fourier transform infrared spectroscopy studies reveal the presence of the amine group at the surface of silica nanoparticles. The silica nanoparticles exhibit blue fluorescence with an emission maximum of 454 nm at 370 nm excitation and show excitation-dependent emission properties in the aqueous medium. With the perfect spectral overlap between silica nanoparticle emission (donor) and chlorophyll absorption (acceptor), fluorescent silica nanoparticles enhance plant photosynthesis rate by resonance energy transfer. This process accelerates the photosynthesis rate to improve the individual plant’s quality and growth. These findings suggested that the fly ash-derived functionalized silica nanoparticles could be employed as nanofertilizers and novel delivery agents.

## Introduction

Chemical fertilizers are employed excessively by farmers in developing countries to achieve increased agricultural productivity, ranging from 50 to 55 percent. These fertilizers range from organic to inorganic and are used by farmers excessively to achieve desired productivity. However, the effectiveness of used nutrients added by these fertilizers is still relatively poor^1,2^. Using excessive nutrients also damages the health of the soil, environment and water world. Moreover, the uncontrolled use of chemical fertilizers increases cultivation costs and decreases the profit of farmers^3–6^. Nanotechnology is rapidly used to create nanosized particles with outstanding qualities, including higher surface area and improved optical and physiochemical properties^7–17^, which can increase farming production in a new way.

Nanofertilizers can provide sufficient nutrients to the plants to promote their development and production. The gradual pattern of nutrient release from nanofertilizers reduces nutrient losses, improving nutrient utilization efficiency. Nanofertilizers are essential for sustained agricultural productivity with high water holding capacity, demand-driven-smart patterns for releasing nutrients, and environmentally pure qualities^18,19^. They have a high surface-to-volume ratio, high sorption capacity, controlled release kinetics to targeted sites, and many active sites for biological activity^20^. Ha et al. prepared NPK nanofertilizer by loading nitrogen (N), phosphorous (P) and potassium (K) into chitosan nanoparticles, synthesized via ionic gelation of tripolyphosphate and chitosan solution^21^. Tarafder et al. proposed a new formulation of a hybrid nanofertilizer in which a calcium hydroxide and orthophosphoric acid mixture were used to fabricate the nitrogen, calcium, and phosphate-rich urea-modified hydroxyapatite nanofertilizer. These mixed nanofertilizers help for slow and sustainable release of nutrients into soil and water^22^. Ekanayake et al. developed a dual-functionalized nanofertilizer capable of releasing micronutrients while nourishing its surrounding soil. The dual-functional nanofertilizer was synthesized from ZnO and CuO nanoparticles^23^.

Recently, fluorescent nanomaterials have been used as a nanofertilizer to enhance plant growth due to their absorption in the ultraviolet region. Fluorescent nanomaterials absorb ultraviolet radiation of sunlight and fluoresce in the visible part, whereas green plants mainly absorb sunlight radiation in the visible region due to chlorophyll. Therefore, there is a strong possibility of energy transfer from the nanomaterials to the plant’s chlorophyll, which increases the rate of photosynthesis tremendously via fluorescence resonance energy transfer process^24,25^. Saikia et al. developed coal-derived functionalized fluorescent carbon quantum dots as a nanofertilizer to promote plant growth^26^. Liang et al. reported fluorescent carbon dots as nanofertilizers that help grow mung plants and this team further explored the fluorescent material for the cell imaging of mung plants^27^. Tan et al. fabricated nitrogen-doped fluorescent carbon dots as a nanofertilizer for the enhancement of the chlorophyll content as well as the electron transfer rate, which helps in the energy transfer activity of the plant^28^.

Fluorescent Silica nanomaterials (SiNPs) have emerged as a new class of environmentally friendly nanomaterials with particle sizes ranging from 5 to 100 nm. Owing to their one-pot synthesis, water dispersibility (monodisperse), colloidal stability, size control, bright fluorescent, and ease of surface functionalization (mainly conjugated to biological macromolecules), SiNPs are becoming potential candidates for modern technologies^29^. Several intrinsic or extrinsic defect centers are responsible for the fluorescence band. The SiNPs help in the direction of plant growth and grant tolerance to diverse abiotic and biotic stimuli. The uptake of SiNPs by plants has risen due to the nano size, higher absorptivity and surface functionalization. The reactive-oxygen species lipid peroxidation decreases in the accumulation of nanosized SiNPs. The accumulation of nanosized and functionalized SiNPs in the leaves and roots decreases the reactive oxygen species and prevents the entry of sodium ions/other heavy metals into plants. Simultaneously, the use of SiNPs as nanofertilizers helps to protect the plant against pathogens in the leaf tissue^30–33^. More importantly, the sprayed fluorescent SiNPs in the leaves transfer their emission energy to chlorophyll, increasing the photosynthesis rate under sunlight exposure^34,35^.

In this connection, we have chosen fly ash as a waste precursor for preparing fluorescent SiNPs. The fly ash obtained from coal-based thermal power plants mainly contains a substantial amount of silicon dioxide (SiO_2_), aluminum oxide (Al_2_O_3_) and calcium oxide (CaO). The only closest prior art uses a fungal-mediated process (96.0 h) to prepare fluorescent SiNPs from fly ash, wherein the fluorescent property was imparted by protein encapsulation during the bioleaching process^36^.

Therefore, a quick, environmentally benign and cost-effective process is highly warranted for nanofertilizer application. Accordingly, the present study provides a one-step method to make monodispersed, blue-emitting, amine-functionalized SiNPs in an aqueous solution from fly ash. Moreover, this study offers a sustainable practical illustration of how fly ash, a waste and environmental risk product, can be utilized as a wealthy nanofertilizer to promote plant growth using fluorescence property.

## Experimental details

### Materials

Fly ash**,** used as a raw material, was collected from coal-fired Vindhyachal thermal power plants in India. All the reagents used in this research work were analytical grade and used without further purification. Sodium hydroxide and triethylamine were purchased from RANKEM and Sigma Aldrich, respectively. Double distilled water was used throughout the research experiment.

### Instrumentation

The particle size distribution was measured through transmission electron microscopy (TEM), FEI-Tecnai G2 12 Twin 120 kV TEM at 120 kV accelerating voltage. The scanning electron microscope (SEM) and energy dispersive X-ray spectroscopy (EDS) analysis have been carried out using Zeiss Merlin VP compact. For the surface functionality characterization and atomic percentages with electronic configurations of presented elements, an experiment was carried out using X-ray photoelectron spectroscopy in PHI 5000-versa probe III instruments. The counts per second of different functional groups as a function of binding energy(eV) were measured using a standard monatomic argon ion gun capable of generating 5 eV to 5 keV Ar ion beams. X-ray diffraction analysis was performed using the Rigaku Smartlab multipurpose high-resolution x-ray diffractometer (HRXRD) automated with the guidance of SmartLab Studio II software to determine either the lattice parameters or the arrangement of individual atoms in a single crystal. A fluorescence study has been carried out using highly sensitive Horiba PTI Quantamaster™ 400, an open architecture steady-state fluorescence spectrophotometer with a continuous 75W xenon arc lamp. The UV–vis electronic absorption spectra measurements were carried out in an Agilent Carry 5000 UV–Vis-NIR spectrophotometer with a maximum scanning speed of 2000 nm/min and a spectral bandwidth of 0.01 nm to 5.00 nm at the wavelength of 175 nm to 3300 nm.

### Synthesis of fluorescent silica nanoparticles from fly ash

**Figure 1:**
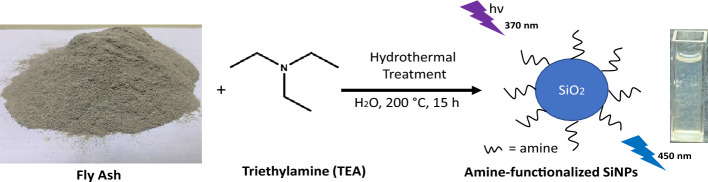
Schematic representation for preparing amine-functionalized fluorescent SiNPs from fly ash using hydrothermal treatment.

The fine powder of coal fly ash (1.5 g; < 72 mesh) was mixed with 25.0 mL of double distilled water and with 5.0 mL of triethylamine (TEA). The mixture was autoclaved at 200 °C for 15.0 h in a 50 mL Teflon-lined container (hydrothermal reactor). After the reaction time, the hydrothermal reactor was cooled down naturally. The obtained mixture was filtered and centrifuged at 10,000 rpm for 10 min. The supernatant portion was purified using a 1.0 kDa (MW) cut-off dialysis membrane for 24.0 h in distilled water, allowing the removal of unreacted small molecules and metals. The pH of purified SiNPs (7.0 mg/mL) was maintained at around 6.6 in distilled water and kept at 4 °C for further use. The product yield of prepared water-dispersed SiNPs was calculated at 4.09%. The schematic protocol for preparing fluorescent SiNPs in an aqueous medium is shown in Fig. 1.

### Experimental design and sampling

The pot experiment was performed in a randomized block design with six treatments (two doses X three types of plants) and three controls (for three kinds of plants) in triplicate. The *O. sanctum* (CIM-Ayu) (family Lamiaceae) is used in traditional medicine, *A. paniculate* (family Acanthaceae) is used in ayurvedic medicine*,* and *S. lycopersicum L*. (family Solanaceae) is a highly consumed vegetable. These plants were taken for the study to determine the effect of SiNP formulation on essential oil yield, secondary metabolite content and fruit yield, respectively. The nursery of the plants was taken from CSIR-CIMAP, an experimental farm. One-month-old plantlets were transplanted to the pots (3 kg filled sandy loam soil) and placed in the greenhouse. The soil was collected from the field (CSIR-CIMAP Lucknow) and classified as a sandy loam texture (Entisols) having 19.0% clay, 19.0% silt and 66.5% sand^37^. After the stabilization of plantlets (15 days), the SiNP formulation was applied (30 mL) at two different concentrations (75 mg/L and 150 mg/L). For the formulation application, 300 mL hand spray bottle was used with a cone nozzle for uniform distribution of nanofertilizer^38^. The application rates of SiNP formulation were taken according to previous reports^26^ and to avoid agglomeration. A Spray of SiNPs formulation was done five times (150 mL of formulation was added for each dose). The plants were exposed to 16 h of light, and the temperature was maintained at 27/22 ± 2 °C Day/night, with an indoor air humidity of 65–80% as demonstrated previously for the foliar application of nano-silica, silicon, and salicylic acid^39,40^.

The plant was grown for three months, and the fresh leaf samples were collected from the third and fourth internode to test its enzymatic activity and growth parameters. For yield, the plants were harvested and rinsed with deionized water, and the new weight and length of the plants were determined. The collection of plant material complies with relevant institutional, national, and international guidelines and legislation.

### Determination of plant parameters

Twelve weeks after spray, the individual plants were divided into root, shoot, and leaf sections. A steel ruler measured the plant height and root length. Fresh weights were determined using a digital balance, and the total chlorophyll concentration in fresh leaves samples was measured following the process described by Arnon and Whatley^41^. The Protein content in plant leaves was estimated according to the Lowry method^42^. The activity of stress-induced antioxidant enzymes such as superoxide dismutase (SOD) and catalase (CAT) was examined by the approach outlined by Kakkar et al., 1984 and Luck et al., 1974 respectively^43,44^, and the proline content was estimated via the procedure presented by Bates et al., 1973^45^. The fresh leaves samples of *O. sanctum* (CIM-Ayu), *A. paniculata,* and *S. lycopersicum L*. were homogenized in a mortar pestle in ice-cold conditions with 2.0 mL of extraction buffer solution [50 mM phosphate buffer (pH 7.8), 2.0 mM EDTA, 5 Mm β-mercaptoethanol and 4% Polyvinylpyrrolidone (PVP)]. The homogenate was then centrifuged for 15 min at 10,000 rpm at 4 °C. Then the stress-induced antioxidant enzymes as catalase (CAT) and superoxide dismutase (SOD) were determined in the supernatant spectrophotometrically. Catalase activity was done by the method previously reported (Luck, 1974). The supernatant (0.1 mL) was added to the reaction mixture containing 3 mL of H_2_O_2_ (30 mM) and 0.01 M phosphate buffer (pH 7.0) and the absorbance was measured at 240 nm. Superoxide dismutase (SOD) was done by the method previously reported by Beauchamp and Fridovich (1971)^46^. The assay mixture contained sodium phosphate buffer (pH 7.8, 50 mM), 1.72 mM Nitroblue tetrazolium (NBT), 201 mM Methionine, 1% Triton X-100, extract of leaves, and 0.12 mM Riboflavin. The absorbance of the reactions was recorded at 560 nm. The proline content was estimated via the procedure presented by Bates et al., 1973^45^. The fresh leaves samples were homogenized with 3.0 mL of sulfosalicylic acid (3%) (in ice-cold condition). The supernatant (2 mL) was reacted with 2 mL of ninhydrin and 2 ml of glacial acetic acid in a test tube for 1 h at 100 °C (reaction terminated in an ice bath). Then the reaction mixture was extracted with 4 ml of toluene (mixed vigorously with a test tube stirrer for 30 s). The chromophore containing toluene was aspirated from the aqueous phase and the absorbance was measured at 520 nm.

### Metabolite analysis

To analyze plant metabolites, shade-dried and ground leaves (100 mg) were added to 10 mL of methanol and then sonicated for 30 min. This process was repeated three times and then dried by a rotary evaporator. After that, 1 ml of methanol (HPLC grade) was added and filtered through a PVDF 0.22 µm syringe and analyzed by High-performance liquid chromatography (HPLC) (Shimadzu model SPD-M20A, Japan)^47^.

### Analysis of the essential oil

The constituents of *O. sanctum* oil were identified using GC and GC–MS analysis. The GC was performed Agilent 7890 B equipped with column ELITE-5 (30 m × 0.25 mm and 0.25 µm) with autosampler (7693). The carrier gas was hydrogen at a 1 ml/min flow rate with a split ratio of 1:150. The injector and detector temperature were set at 290 °C. The oven was programmed from 60 to 246 °C at 3 °C/min. The GC–MS was performed GC interfaced (PerkinElmer AutoSystem XL) with a Turbomass Quadrupole mass spectrometer^48^.

### Extraction of pigments

To determine lycopene and β-carotene, 1.0 g of liquidized pulp was added to the solvents (10 mL of ethanol, 10 mL of acetone, and 20 mL of hexane). The mixture was placed in a tube covered with silver foil and a lid and stored in a refrigerator at 3 °C, where it remained until complete depigmentation. Then again, 10 mL of ethanol, 10 mL of acetone, and 20 mL of hexane were added; this was later filtered in Whatman filter paper number 8; after that, 50 mL of distilled water was added for phase separation, and then the whole mixture was poured into a 150 mL test tube, discarding the previous fraction. The absorbance was recorded at 503 nm for the lycopene and 450 nm for the β-carotene.

### Available nutrient content

The pH and electric conductivity (EC) of soil was analysed using Seven Go Duo TM SG23 pH meter and water holding capacity (WHC) of soil was determined by using standard methods^49^. Soil organic carbon was determined by oxidation of dichromate and titration with ammonium ferrous sulphate^50^. Exchangeable NH_4_-N and NO_3_-N in the soil sample were determined by extraction in KCl as a method reported by Keeney and Nelson^51^. Available phosphorus in the soil sample analysed using the method reported by Bray and Kurtz^52^. For the total nutrient content soil was digested in biacid solution. However, the soil was extracted DTPA (diethylenetriamine pentaacetic acid) for quantification of available nutrients (Al, Fe, Ca, Co, Cu, K, Mg, Mn and Ni) and then analysed by inductively coupled plasma (ICP OES, Perkin Elmer, Optima 5300 V). The silica content was analysed using scanning electron microscopy (SEM)-EDX (JSM6100, Make: JEOL USA)^38^. The available inorganic sulphur in soil was determine by the method given by Butters and Chenery^53^.

## Results and discussion

### Characterization of amine-functionalized fluorescent SiNPs

#### Morphological characterization

**Figure 2:**
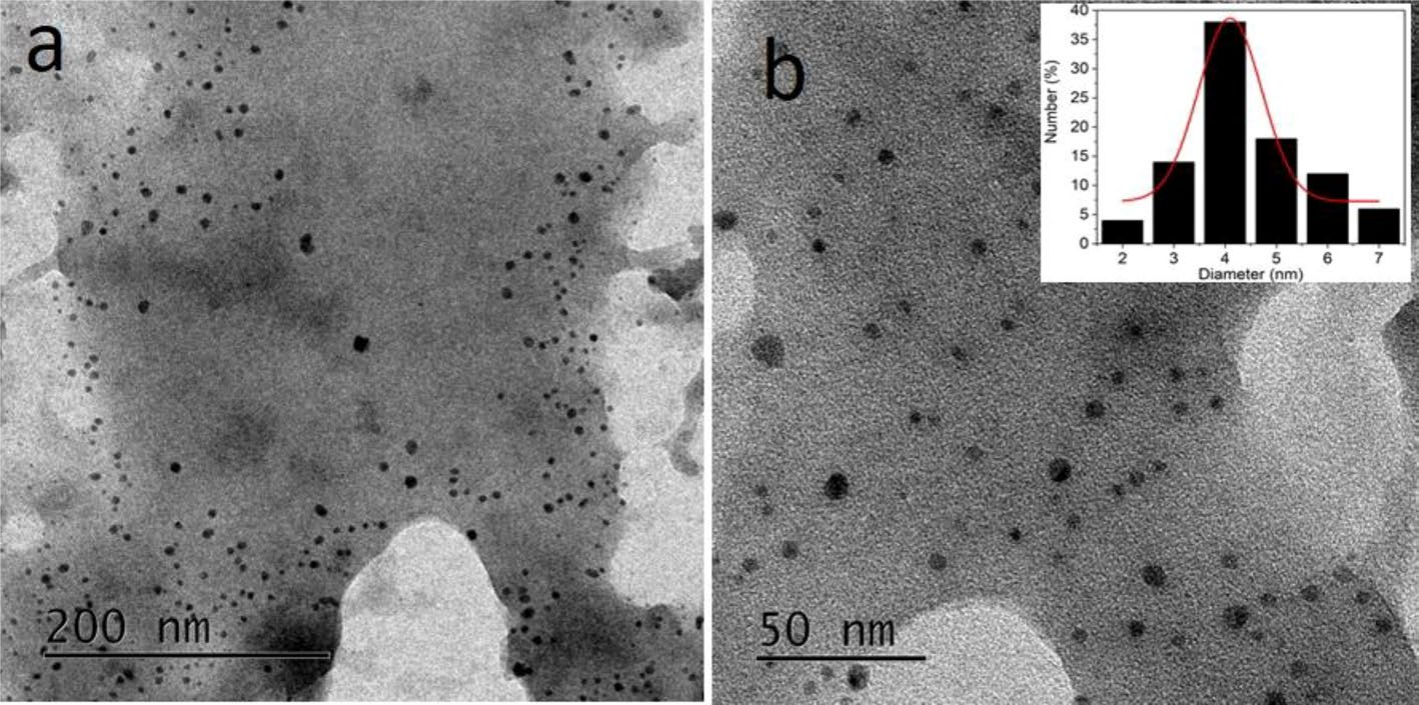
(**a**) Low and (**b**) High magnification TEM image of fly ash-derived SiNPs.

Transmission electron microscope (TEM) analysis was carried out to understand the size and morphology of SiNPs. The low and high magnification images of SiNPs are shown in Fig. 2a,b, which shows that the SiNPs have a spherical morphology and are well dispersed in the aqueous medium. The average diameter of SiNPs is calculated from the particle size distribution plot (inset, Fig. 2b) and found to be 4.0 nm.

**Figure 3:**
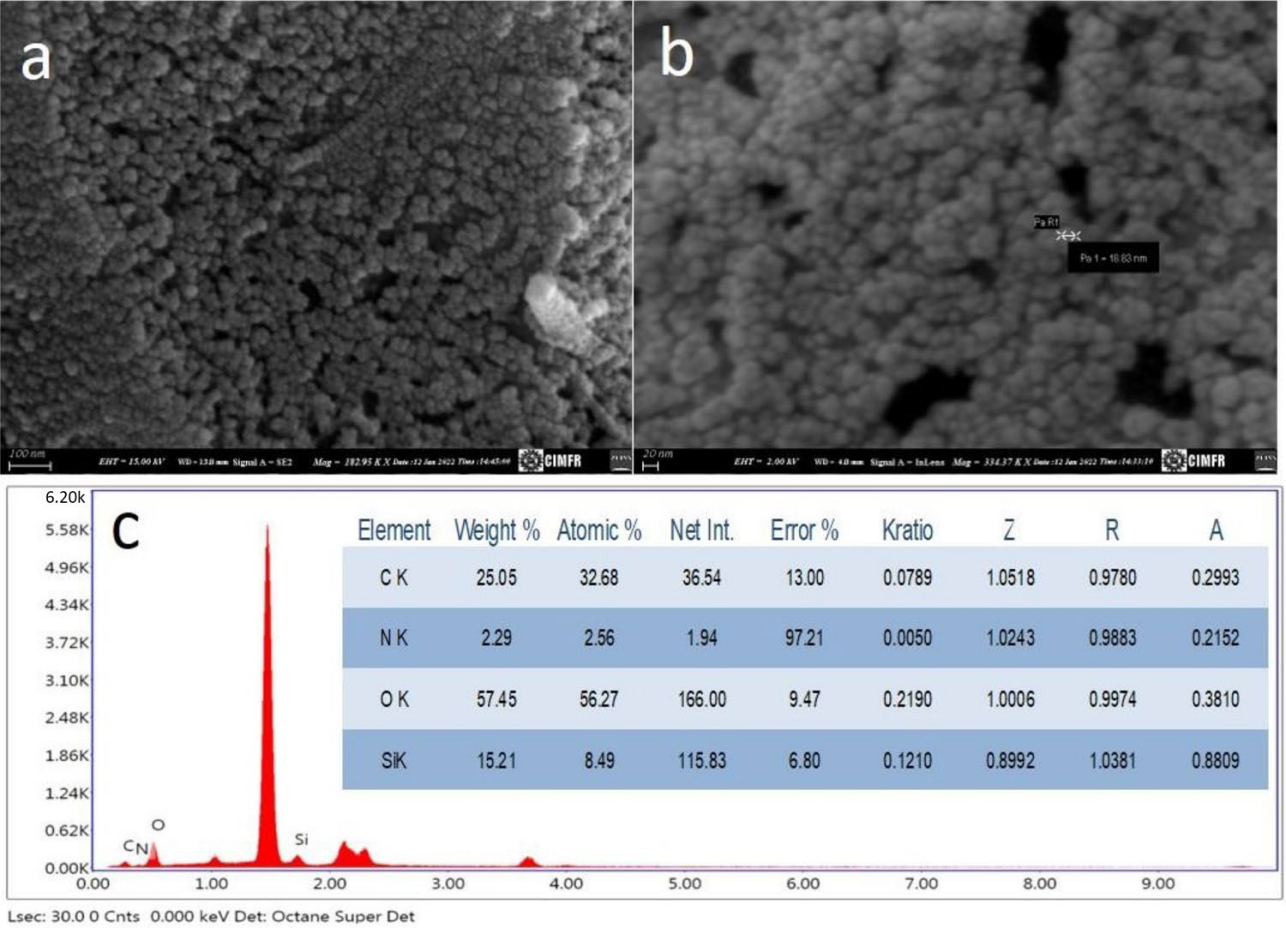
(**a**) Low, (**b**) High magnification FESEM images and (**c**) EDS spectrum with the elemental percentage of fly ash derived SiNPs (inset).

Field emission scanning electron microscopic (FESEM) studies have been done to understand the internal structure and morphology of SiNPs. The low- and high-magnification FESEM images are shown in Fig. 3a,b, respectively. It can be found in Fig. 3a that these nanoparticles are uniformly arranged and have spherical morphology. Spherical-shaped particles with little agglomeration were observed in Fig. 3b. The energy dispersive spectroscopy (EDS) analysis was used to determine the chemical composition of the SiNPs. The significant elements' peaks were silicon, carbon, oxygen, and nitrogen in the EDS spectra (Fig. 3c), indicating that SiNPs are functionalized with an amine. The presence of aluminum in EDS was due to using aluminum foil for SEM analysis. The EDS study confirms the SiNP formation as silicon and oxygen constituents weigh more than 70% of nitrogen and carbon (inset Fig. 3c).

**Figure 4:**
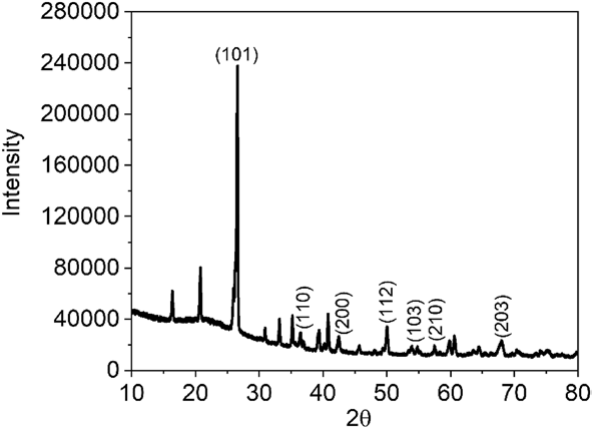
X-ray diffraction pattern of SiNPs with the indices for the crystalline SiNPs.

The XRD pattern shows distinct diffraction peaks in the 2θ range of 20–80° in Fig. 4. The synthesized SiNPs have the characteristics peaks at (101), (110), (200), (112), (103), (210) and (203). These peaks represent the purity and crystalline nature of the silica nanoparticles. It is important to note that even though the SiNPs are functionalized with the amine group, the presence of the amine group does not compromise the crystallinity of the SiNPs.

**Figure 5:**
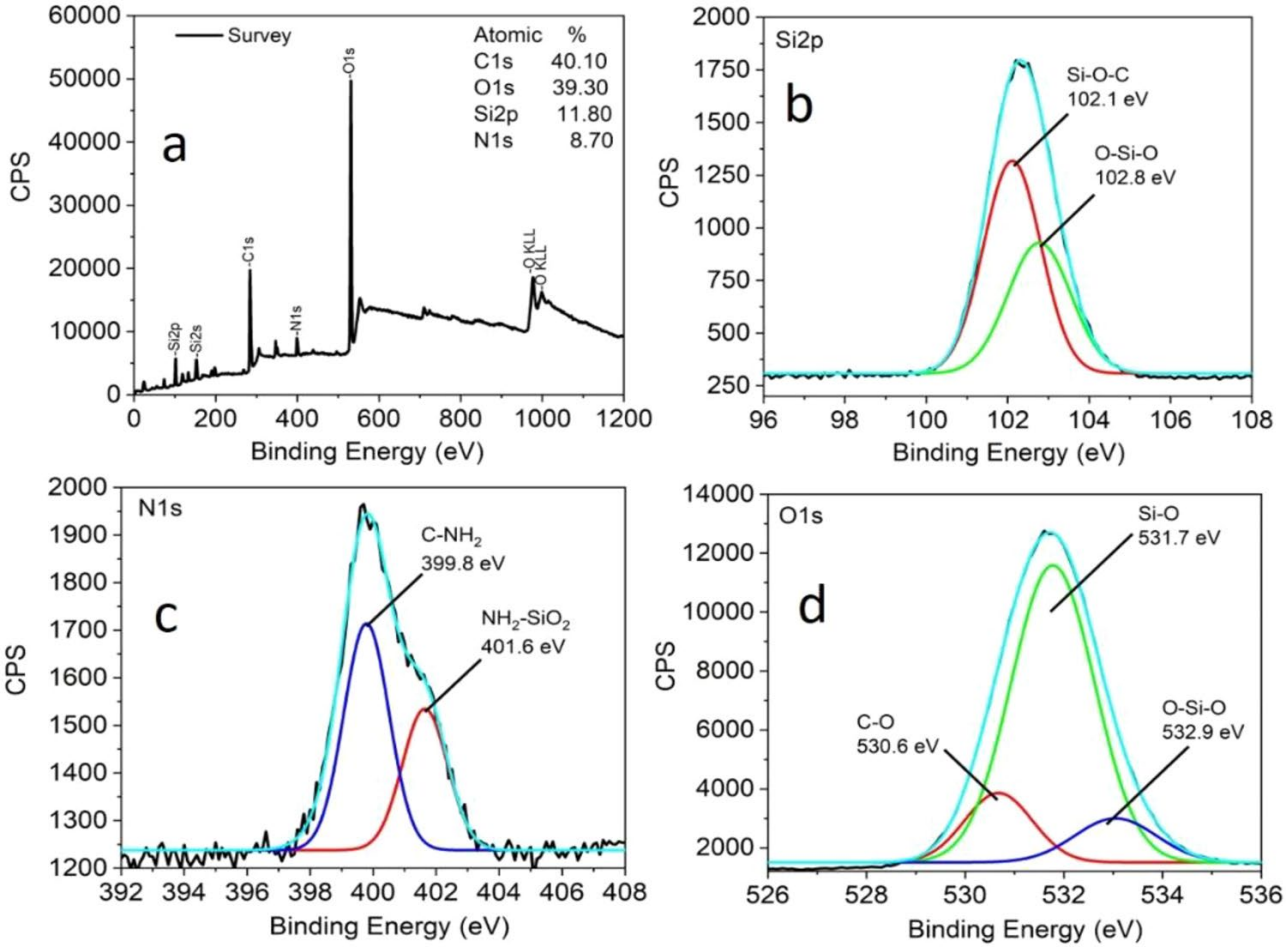
(**a**) Full range XPS spectral profile; deconvoluted high-resolution XPS spectra (**b**) Si2p region, (**c**) N1s region and (**d**) O1s region of SiNPs.

X-ray photoelectron spectroscopy (XPS) and FT-IR analyses were carried out to understand the nature of surface functional groups in SiNPs. The wide XPS scan shows signals due to the presence of carbon (40.10%), oxygen (39.30%), silicon (11.80%) and nitrogen (8.70%). The full XPS spectrum of SiNPs with five prominent peaks at 101.5, 152.4, 284, 399, and 531 eV are attributed to Si2p, Si2s, C1s, N1s and O1s, respectively (Fig. 5a). The result suggested that the SiNPs mainly comprise the elements Si, C, N and O. The binding energy values of particular elements were obtained from a high-resolution XPS spectrum. Figure 5b shows the full XPS spectrum of Si2p, which has two different chemical environments, corresponding to O–Si–O at 102.8 eV and Si–O–C at 102.1 eV. Significantly, the observed high binding energy for SiO_2_ compared to SiOC is due to the boding of Si atoms with two electronegative oxygen atoms (electron withdrawing power), leading to a decrease in the electron density (increase of effective nuclear charge) of the 2p orbital electron in Si atom. Therefore, the electron holding capacity of 2p orbital in the SiO_2_ increases, and higher energy is required to release an electron in XPS analysis. Two peaks observed at 399.8 and 401.6 eV correspond to C–NH_2_ and NH_2_–SiO_2_, respectively, in N1s XPS spectrum (Fig. 5c). The binding energy values for O1s observed at 530.6, 531.7 and 532.9 eV are attributed to C–O, Si–O and O–Si–O, respectively (Fig. 5d). Various functional groups are found in SiNPs from FTIR spectra (Supporting Information; Fig. S1). The SiNPs show peaks at 3746 and 1700 cm^-1^ due to the stretching and bending vibration of the N–H bonds present. Similarly, peaks at 1522, 1090, 912 and 790 cm^-1^ correspond to C–N stretching, Si–O–Si asymmetric stretching vibration, Si–O characteristic stretching peak, and Si–O–Si symmetric stretching vibration. The peak at 2358 cm^-1^ is due to the CO_2_ background. The XPS and FTIR studies show that the SiNPs are functionalized with the amine group.

#### Photophysical study

**Figure 6:**
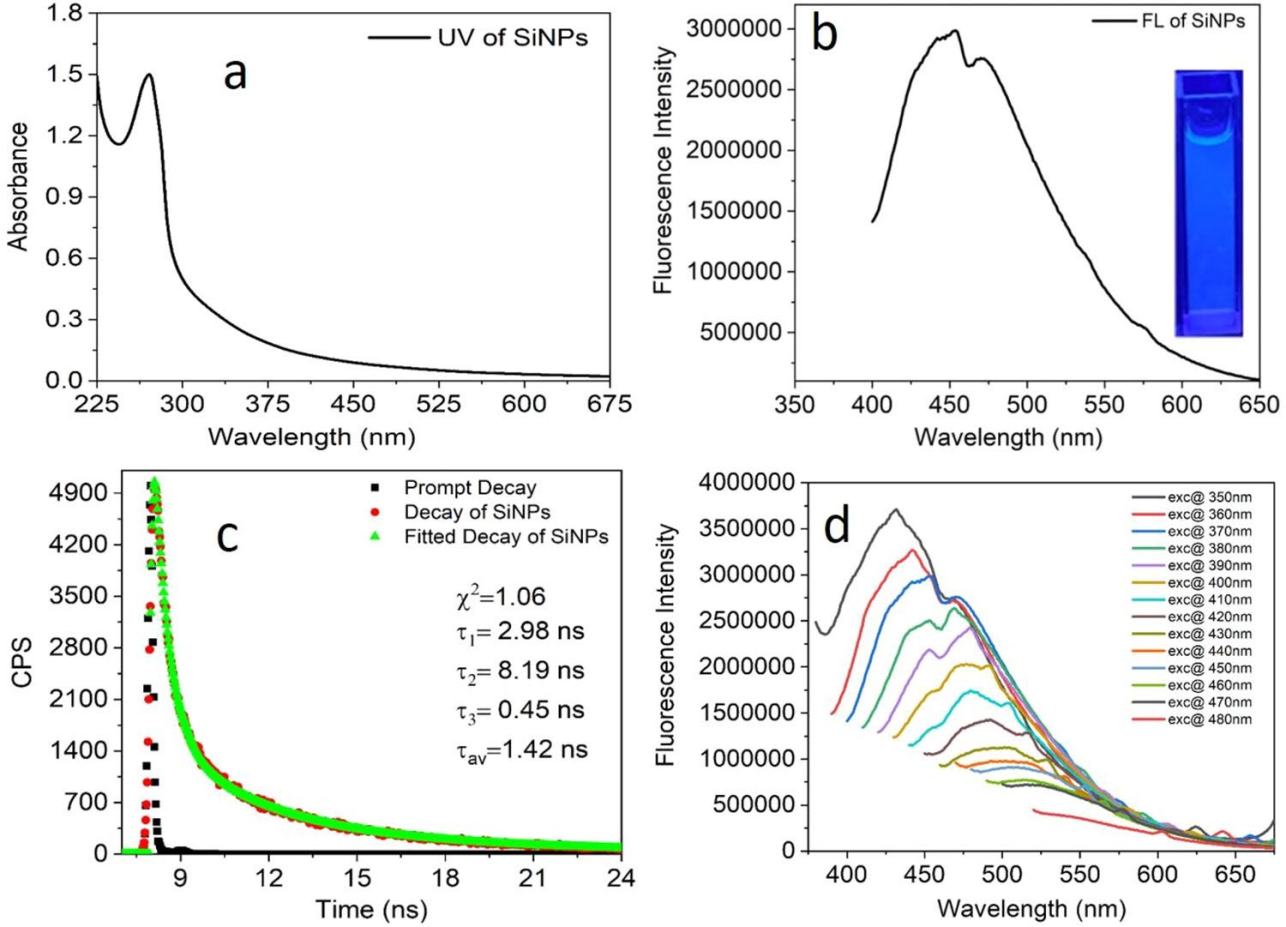
(**a**) UV–visible absorption, (**b**) fluorescence emission spectrum (Inset; fluorescent image under UV light exposure) of SiNPs; λ_exc_ = 370 nm, (**c**) Triexponential fitted fluorescence decay plot and (d) Excitation-dependent emission spectra in aqueous medium [SiNPs] = 7.0 mg/mL.

The UV–Vis absorption spectrum of a clear transparent neutral aqueous solution of amine-functionalized SiNPs (7.0 mg/mL) has a prominent absorption peak at 271 nm and an absorption edge at 338 nm due to the intrinsic bandgap of SiNPs^54^ (Fig. 6a). The excited states of the SiNPs at 271 nm and 338 nm corresponding to the dioxasilyrane (= Si(O_2_)) and silylene (Si) defects pairs. Since the concentration of amine-functionalized SiNPs cannot be calculated accurately, the optical density of the SiNPs was kept in the range of 0.2–0.7 at the excitation wavelength to avoid the inner filter effect. The transparent solution of SiNPs showed blue fluorescence with an emission maximum of 454 nm at 370 nm excitation (Fig. 6b). The blue fluorescent image of SiNPs in an aqueous medium under UV light illumination has been shown in the inset, Fig. 6a. The characteristic absorption bands and the emission spectra of the SiNPs indicated that the fluorescence originated from the surface defect pairs (= Si(O_2_)) and silylene (Si)) in silica. The hydrothermal treatment of the SiNPs increased the surface defect pairs and suppressed the non-emissive thermal decay, resulting in fluorescent SiNPs^55^. It was observed that SiNPs give weak fluorescence signals without amine functionalization and almost 15 times enhancement of fluorescence intensity of SiNPs in the presence of TEA (supporting information; Fig. S2). The relative quantum yield of SiNPs (without amine) and amine-functionalized SiNPs has been found at 14% and 35% by taking quinine sulfate dye as a standard. The enhancement quantum yield of amine-functionalized SiNPs is due to (i) the prevention of agglomeration of SiNPs in the aqueous medium by the amine group and (ii) the electron donation nature of the amine group stabilized the electron-deficient silylene defect^54,55^. The enhanced emission intensity of amine-functionalized SiNPs in the visible range helps the plant reabsorb the light by photosystem II (PS II) via resonance energy transfer. Hence, the amine functionalization and blue fluorescence increased the nutrition of the plant, as well as accelerate the rate of photosynthesis. As a result, the growth and quality of plants enhanced.

The lifetime decay of the fluorescence emission was studied at 405 nm excitation wavelength with 490 nm emission wavelength. The lifetime decay curve was fitted to the data using triexponential decay models, resulting in the optimal fit with χ^2^ value 1.06. The average lifetime for the SiNPs was 1.42 ns in aqueous medium (Fig. 6c).

The excitation-dependent emission spectra of amine-functionalized SiNPs are an intrinsic property of the SiNPs. To explore the excitation-dependent emission property of SiNPs, we scanned the samples from 350 to 480 nm excitation wavelength (with 10 nm interval) and collected their emission response from 370 to 700 nm (Fig. 6d). A redshift in the emission profile was observed. Fluorescence excitation spectra (supporting information; Fig. S3) of SiNPs showed an excitation peak at 360 nm at 442 nm of emission wavelength. The fluorescence excitation spectrum gives information about the electron distribution of the materials in the ground state.

### Silica nanoparticles as nanofertilizer

#### Plant height, biomass, and growth parameters

The pH, EC, organic carbon, and water holding capacity were observed at 8.2 ± 0.1, 61 ± 2 mS, 31.7 ± 0.5 μg/kg, and 30 ± 0.9% respectively. The total minerals in soil were recorded such as Al (3949 ± 145 mg/kg), Fe (12,800 ± 289 mg/kg), Ca (6088 ± 341 mg/kg), Co (2.4 ± 0.6 mg/kg), Cu (4 ± 0.5 mg/kg), K (1300 ± 49 mg/kg), Mg (3659 ± 79 mg/kg), Mn (106 ± 21 mg/kg) and Ni (4.2 ± 0.5 mg/kg)^56^. The available mineral content in soil was recorded such as Al (1.01 ± 0.1 mg/kg), Fe (5.26 ± 0.4 mg/kg), Ca (28 ± 2.1 mg/kg), Co (0.018 ± 0.001 mg/kg), Cu (0.31 ± 0.01 mg/kg), K (13 ± 1.1 mg/kg), Mg (102.6 ± 1.1 mg/kg), Mn (3.9 ± 0.1 mg/kg) and Ni (0.010 ± 0.002 mg/kg), SO_4_ (0.011 ± 0.03 mg/kg). The silica content in soil was 22.78%. Exchangeable NH_4_-N, NO_3_-N and available phosphorus contents in soil were 6.9 ± 0.12 mg/kg, 5.9 ± 0.08 mg/kg and 1.6 ± 0.05 mg/kg.

**Figure 7:**
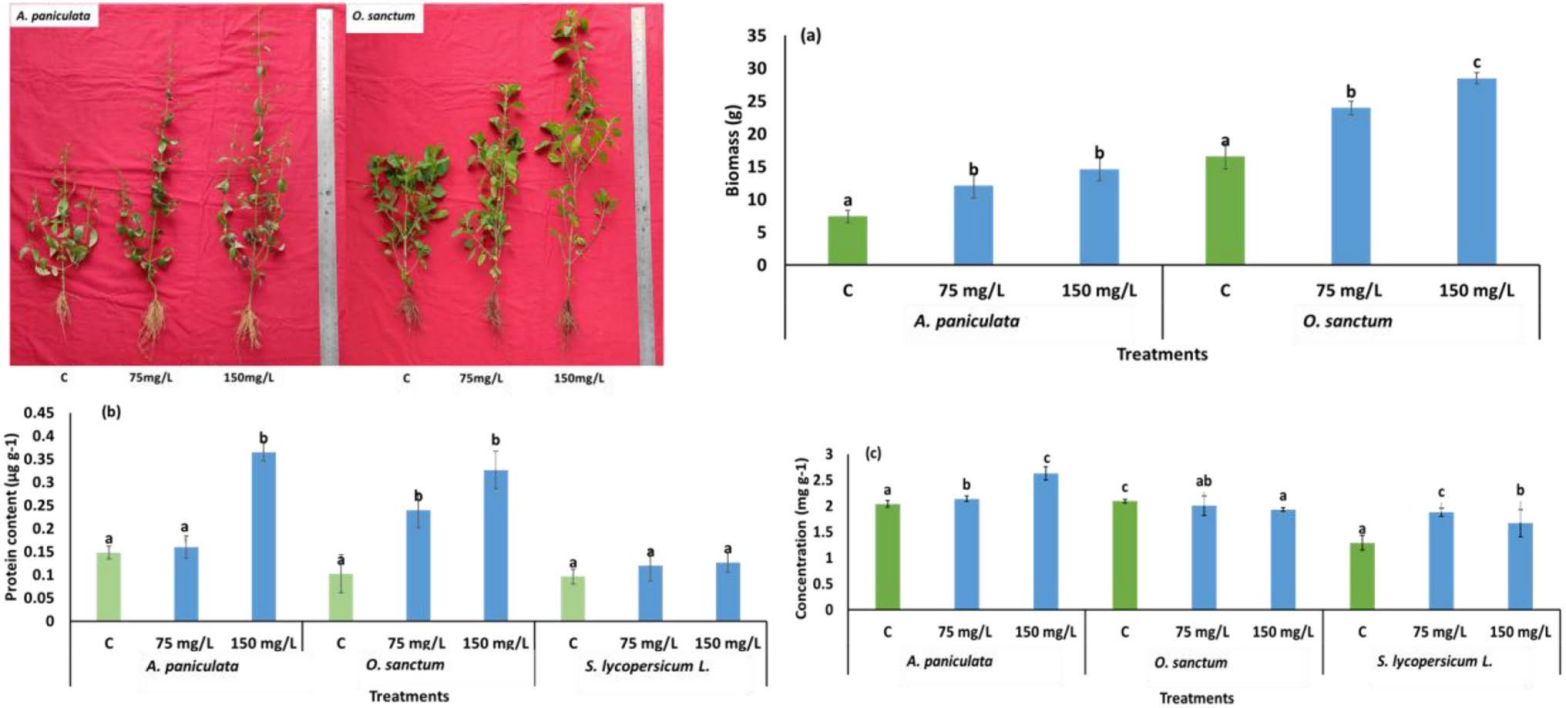
Effect of SiNPs on (**a**) Biomass, (**b**) Protein content, (**c**) Chlorophyll content.

The SiNP-based nano-fertilizer positively affects plant height and biomass. Both the height and biomass of each of the three plants, *O. sanctum* (CIM-Ayu), *A. paniculata,* and *S. lycopersicum* L., are significantly higher than the control, as shown in Fig. 7. The amendment of SiNPs promoted the plant height compared to the control; a higher concentration of SiNPs was more effective in increasing the height of *A. paniculata* as compared to the lower concentration and control. *O. sanctum* and *S. lycopersicum L.* showed the best height at lower concentrations. Both concentrations showed a positive result in increasing plant biomass in *O. sanctum, with* no significant difference between higher and lower concentrations. However, *A. paniculata* biomass data showed a substantial increase at a higher concentration than the lower concentration and control. SiNPs act as growth promoters and increase organic compounds^57^ (Suriyaprabha R et al., 2014), enhancing water uptake capacity^58^ (Janmohammadi M. et al., 2016), as evidenced by many studies. Fitriani and Haryanti (2016) found in their research that different concentrations of silica nanoparticles promoted plant height, leaf number, and root length of *S. lycopersicum*^59^. Yassen et al., 2017 also observed that foliar spray of silica nanoparticles at different concentrations (0, 15, 30, 60, and 120 mg/L) increased the growth, yield, and chemical composition of cucumber (*Cucumis sativus*)^60^. Figure 7 shows treated *A. paniculata* and *O. sanctum* plants have significantly higher protein content*. *In *A. paniculata, *only a higher concentration of SiNPs increased 166.6% of protein content was observed, and a lower concentration showed a non-significant increase (14.2%) as compared with the control. In *O. sanctum*, both the higher and lower concentrations significantly increased the protein content (226.2% and 139.5%), respectively, and *S. lycopersicum L.* non-significantly increased (33.1% and 40.7%). Hellala, F. et al., 2020 also reported an increment in protein and chlorophyll content in barley due to the application of nano Silica^61^. In our study, the chlorophyll content of *A. paniculate* and *S. lycopersicum L. *significantly enhanced at 75 mg/L-6.9%, 150 mg/L-31.3%, and 75 mg/L-56.7%, 150 mg/L-39.0%, respectively. At the same time, a significant decrease (75 mg/L-0.33%; 150 mg/L-3.5%) in chlorophyll content was observed in the *O. sanctum.* These studies showed that only *A. paniculate and S. lycopersicum L.* had increased chlorophyll content due to the SiNP application; *O. sanctum* showed no such improvement*.* As per the reported studies, silica dioxide nanoparticles improved photosynthesis, photochemical efficiency, electron transport rate, PSII activity, net photosynthetic rate, transpiration rate, stomatal conductance, and synthesis of photosynthetic pigments^62,63^ which ameliorate the growth and development of plants.

#### Stress enzymes and plant metabolite content

The activity of major antioxidants proline (0.003–0.019 units/mg protein/min), CAT (0.017–0.143 units/mg protein/min), and SOD (0.006–0.120 unit/mg FW) were observed in treated plants (Table 1). The data suggest that proline activity in *Andrographis paniculata* was significantly enhanced in treated plants compared to control. This could be the reason treated plants have lower levels of metabolites than the control plants. The concentration of the secondary metabolites of *A. paniculate* varied from 0.002 to 2.24% for andrographolide, from 0.001 to 1.67% for neo andrographolide, from 0.0003 to 0.16% for DDA (14-deoxy-11,12-didehydroandrographolide) and 0.001–0.029% for andrographanin (Fig. 7). In several studies it was reported that the toxic effects of SiNPs on plants systems depend upon various factors such as concentration, stability, particle size and synthesis process^64,65^. *Ocimum sanctum* has negligible proline activity at lower concentrations. At the higher dose, it was significantly higher than the control. Whereas in *Solanum lycopersicum L.* was non-significant. No significant difference between control and treated plants for catalase activity was observed in all three. Again, for SOD, no significant difference was observed in *Andrographis paniculata* and *Solanum lycopersicum L*. In *Ocimum sanctum* at a higher concentration, it was significantly decreasing as compared to the control and non-significant at a lower concentration.

**Table 1:** Effect of SiNPs on antioxidants: Catalase, Superoxide dismutase (SOD), and Proline. (In Supplementary).

S. no	Parameters	Treatments	Plants		
			*A. paniculata*	*O. sanctum*	*S. lycopersicum*
1	Catalase	C	0.13 ± 0.01a	0.13 ± 0.01a	0.01 ± 0.009a
		75 mg/L	0.12 ± 0.01a	0.13 ± 0.01a	0.03 ± 0.01a
		150 mg/L	0.14 ± 0.01a	0.13 ± 0.007a	0.04 ± 0.01a
2	SOD	C	0.11 ± 0.003a	0.01 ± 0.001b	0.05 ± 0.009a
		75 mg/L	0.12 ± 0.009a	0.01 ± 0.002b	0.03 ± 0.001a
		150 mg/L	0.10 ± 0.004a	0.006 ± 0.001a	0.02 ± 0.01a
3	Proline	C	0.003 ± 0.001a	0.006 ± 0.001a	0.01 ± 0.002a
		75 mg/L	0.008 ± 0.002b	0.007 ± 0.001a	0.01 ± 0.001a
		150 mg/L	0.015 ± 0.002c	0.010 ± 0.002a	0.01 ± 0.002a

#### The yield of essential oil of Ocimum sanctum

Both doses of SiNPs significantly enhanced the oil yield; the highest was observed at higher (150 mg/L) concentrations. Oil yield was significantly increased by 44% at lower concentrations and 86% at higher concentrations. The effect on the major constituents of essential oil was also observed, and the major difference was found in eugenol. The area percentage for eugenol in control was 6.3%; at lower concentrations 29.7%, and at higher concentrations, 43.4%, indicating the beneficial effects of the foliar application of SiNPs. The other two beta-elemene and caryophyllene, were significantly reduced in treated plants. Beta-elemene in control was 32%; at lower concentrations 25.2%; and higher concentrations, 13%; and caryophyllene in control was 38%; at lower concentrations, 28.8%; and at higher concentrations, 22.8%.

#### Tomato fruit yield

Positive results were observed for fruit productivity; the higher and lower concentrations showed higher fruit productivity and weight than the control. From 28.2 to 156.4%, fruit production increased in treated plants. For lycopene and β-carotene content, no significant difference was observed between control and with SiNPs at both concentrations (supporting information; Fig. S4). In other findings, it was reported that SiNPs might be owed to its role in increasing RNA polymerase expression and the activity of ribosomal proteins, which promote stress tolerance and reduce transpiration rate and oxidative stress, stabilizing the photosynthetic rate and eventually enhance fruit production^66–68^.

#### Sunlight harvesting mechanism

**Figure 8:**
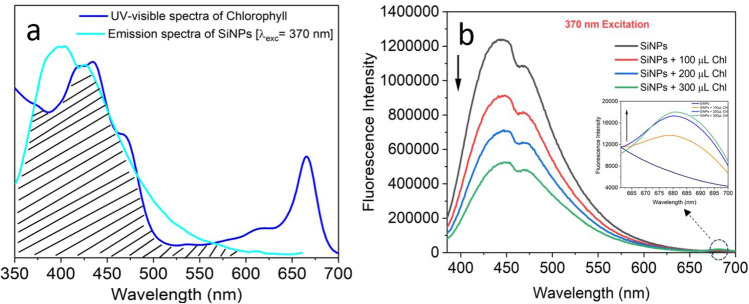
(**a**) Spectral overlap between emission spectra of SiNPs (donor) with UV–visible absorption spectrum of chlorophyll (acceptor) and (**b**) Validation of resonance energy transfer mechanism.

Increasing the effectiveness of photosynthesis and the electron transfer process is considered one of the best strategies for promoting plant development^69^. Due to the perfect spectral overlap of SiNPs emission and chlorophyll (Chl) absorption, there is a strong possibility of energy transfer (Fig. 8a) from SiNPs to Chl. After adding water-dispersed amine-functionalized fluorescent SiNPs into these plants, the fluorescent property of the SiNPs enhances the photosynthesis process in leaves by transferring its emission energy to Chl. We have proved the resonance phenomena by keeping the SiNPs concentration constant while varying the Chl concentration (Fig. 8b). The fluorescent SiNPs absorb UV radiation of the sunlight and emit at visible region (Fig. 8a,b). As a result, it not only increased the rate of the photosynthesis process, followed by enhancing the growth of plants, but also reduced the damage caused by UV radiation to the plants.

This energy transfer phenomenon decreases the fluorescence intensity of SiNPs and rapidly increases the fluorescence intensity at the photosystem II (PS II) region of chlorophyll (inset, Fig. 8b). The rapid electron transmission from SiNPs to chlorophyll directly influences the chain electron transfer pathway in light reactions, hence increasing photosynthesis process.

**Figure 9:**
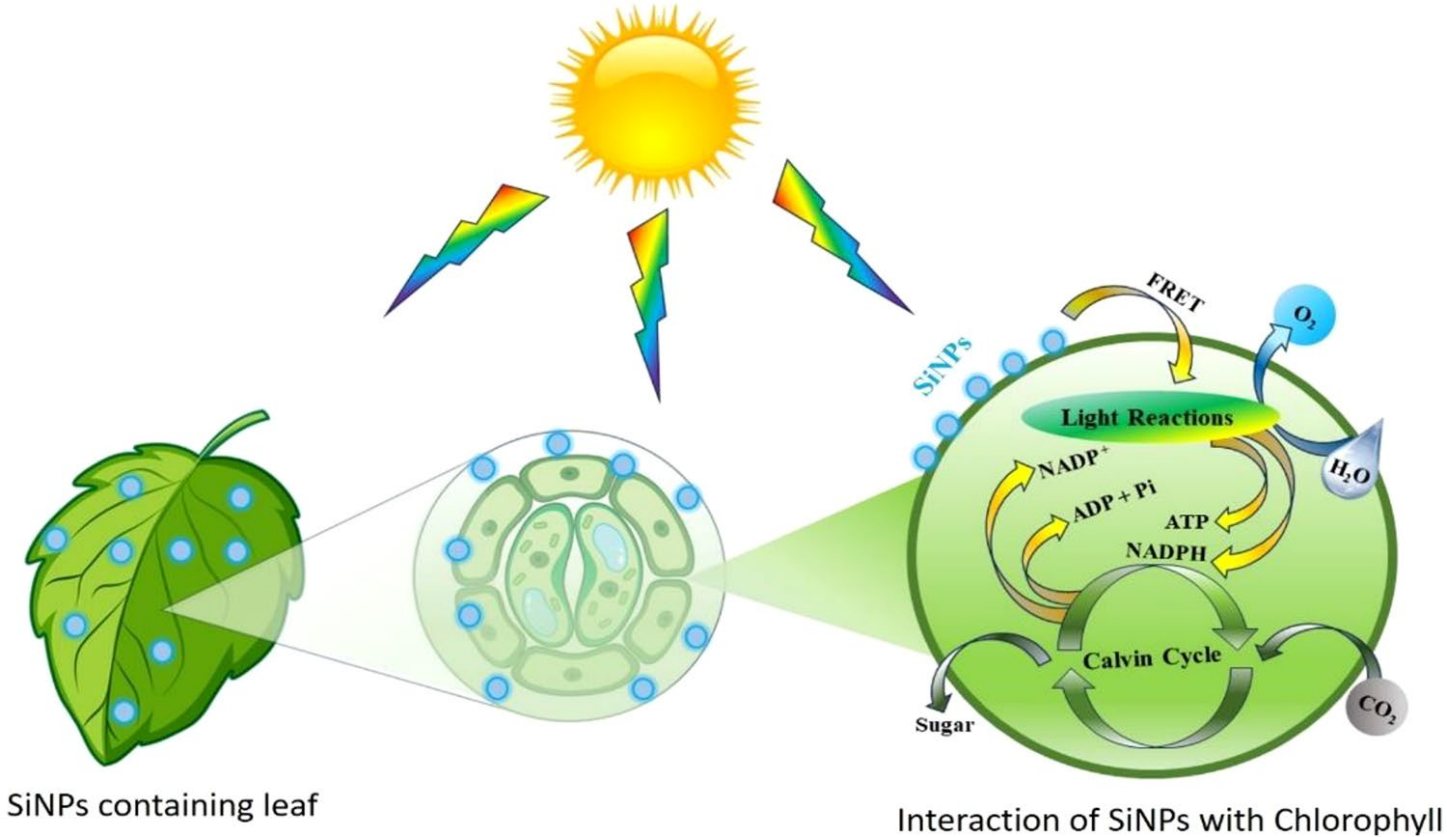
Diagrammatic illustration for plausible sunlight harvesting mechanism.

A possible diagrammatic illustration of the sunlight harvesting mechanism has been shown in Fig. 9, in which SiNPs transfer their energy via the FRET mechanism to plant chlorophyll to enhance the photosynthesis rate. The absorbed energy by chlorophyll helps to split the water molecules into hydrogen and oxygen ions under a light reaction. The oxygen ions recombine to release oxygen gas; on the other side, hydrogen ions react with nicotinamide adenine dinucleotide phosphate (NADP) to form the energy-carrying molecule nicotinamide adenine dinucleotide phosphate hydrogen (NADPH), which is required to produce glucose in the Calvin Cycle. The Calvin Cycle is an essential light-independent reaction step in which the chemical energy in NADPH, adenosine triphosphate (ATP), and carbon dioxide (CO_2_) are used to form glucose.

The precursors for developing the nanofertilizers in the above-cited reports in Table 2 are mainly prepared from costly chemicals and metals. In the present study, we used fly ash as a waste precursor obtained from NTPC, India, to develop fluorescent SiNP-based nanofertilizers. First-time, we explore the fluorescence property of prepared SiNPs to increase the plants' photosynthesis rate via the fluorescence energy transfer mechanism.

**Table 2:** Comparison of the reported nanofertilizers based on their precursors, size and advantages.

Sr. no	Precursors	Nanofertilizer	Key points/advantage	References
1	Copper Sulphate & Zinc Acetate	Zn & Cu Nanoparticles	Rapid absorption & slow release	Abbasifar et al.^70^
2	Citric Acid & Diammonium Phosphate	N-Carbon Dots	Enhancement of lettuce yield and quality	Tan et al.^28^
3	Sucrose & Ortho-Phosphoric Acid	Carbon Nanoparticles	Promote nutrient absorption and accumulation	Shekhawat et al.^71^
4	SiO_2_ & Chitosan	Chitosan-silicon Nanoparticles	Introduce antioxidant-defense enzyme activities and equilibrated cellular redox homeostasis	Kumaraswamy et al.^72^
5	Tetraethyl orthosilicate and Ammonia Solution	Silica (SiO_2_) Nanoparticles	Increase metabolic balance and effective as an insecticide	El-Naggar et al.^31^
6	Sodium silicate, Aluminum sulfate sodium hydroxide Ferrous Chloride & Cupric Chloride	A mixture of Zn-Fe-Cu hybrid nanocomposite and nano zeolite	Increase mineral contents and vitamins	Rahman et al.^73^
7	Zinc acetate dihydrate & citric acid	Zinc Oxide Nanoparticles	Improve the antioxidant defense system	Yusefi-Tanha et al.^74^
8	Sodium citrate, sodium carbonate, urea, potassium phosphate, potassium nitrate and calcium nitrate	NPK-Calcium Phosphate Nanoparticles	Provide and slow release of multi-nutrients to soil	Ramírez-Rodríguez et al.^75^
9	Coal fly ash (waste precursor)	Nitrogen-enrich fluorescent SiNPs	Increase the photosynthesis rate in the plant growth and improve tolerance to the abiotic and biotic stimuli via the FRET mechanism	Current Work

## Conclusions

Water-soluble, crystalline, blue-emitting silica nanoparticles from coal fly ash were reported by employing one-step hydrothermal synthesis followed by amine-functionalization. Their average size and morphological characterization study reveal that SiNPs have an average core diameter of 4.0 nm with spherical morphology and are mono-dispersed in the aqueous medium. The presence of the amine group at the surface of the silica nanoparticles is confirmed by XPS and FTIR analysis. The fluorescence intensity enhanced after functionating with the amine group and exhibited excitation-dependent emission properties in the aqueous medium. The perfect spectral overlap between SiNPs (donor) and chlorophyll (acceptor) helps in the energy transfer process for enhancing the rate of photosynthesis in the plant system to improve plant growth. The soil-applied agricultural nanonutrients have increased by amine functionalization in plant uptake studies, which indicates the potential of SiNPs as nanofertilizers. The gainful utilization of fly ash waste into value-added materials for nanofertilizer applications in agriculture is a tremendous benefit of this approach, resulting in sustainability. The current process is easily up-scalable on a kg scale and reduces the production cost of fluorescent SiNPs compared with available precursors.

### Supplementary Information

Supplementary Information.

## Data Availability

The datasets generated and/or analysed during the current study are available from the corresponding author on reasonable request.
