# Supplementary material for: Development of amine-functionalized fluorescent silica nanoparticles from coal fly ash as a sustainable source for nanofertilizer
Source: Sci Rep. 2024 Feb 6;14:3069. doi: 10.1038/s41598-024-53122-z (PMC10847091; doi:10.1038/s41598-024-53122-z)
Supplement: Supplementary file 1 — Supplementary Information. [file 41598_2024_53122_MOESM1_ESM.docx]

**Supporting Information**

**Development of Amine-functionalized Fluorescent Silica Nanoparticles from Coal Fly Ash as a Sustainable Source for Nanofertilizer**

Vikram Singh^1,2,3^*, Tuhin Mandal^1,3^, Shiv Rag Mishra^1,3^, Anupama Singh^4^, Puja Khare^3,4^

^1^Environment Emission and CRM Division, CSIR-Central Institute of Mining and Fuel Research Dhanbad, Jharkhand, 828108, India

^2^Coal to Hydrogen Energy for Sustainable Solutions, CSIR-Central Institute of Mining and Fuel Research Dhanbad, Jharkhand, 828108, India

^3^Academy of Scientific and Innovative Research (AcSIR), Ghaziabad, 201002, India

^4^Agronomy and Soil Science Division, CSIR-Central Institute of Medicinal and Aromatic Plants, Lucknow 226015, Uttar Pradesh, India

*Corresponding Author: Vikram Singh, Tel: 0326-2388289, Email: [vikku.010@gmail.com](mailto:vikku.010@gmail.com) and vikramsingh@cimfr.nic.in

1. FTIR spectra of SiNPs…………………………………………………Page S1

2. Fluorescence emission spectra with and without amine functionalization……Page S2

3. Excitation spectrum of SiNPs in aqueous medium……………………..Page S3

4. Lycopene and β-carotene content in tomato fruit ..……………………..Page S4


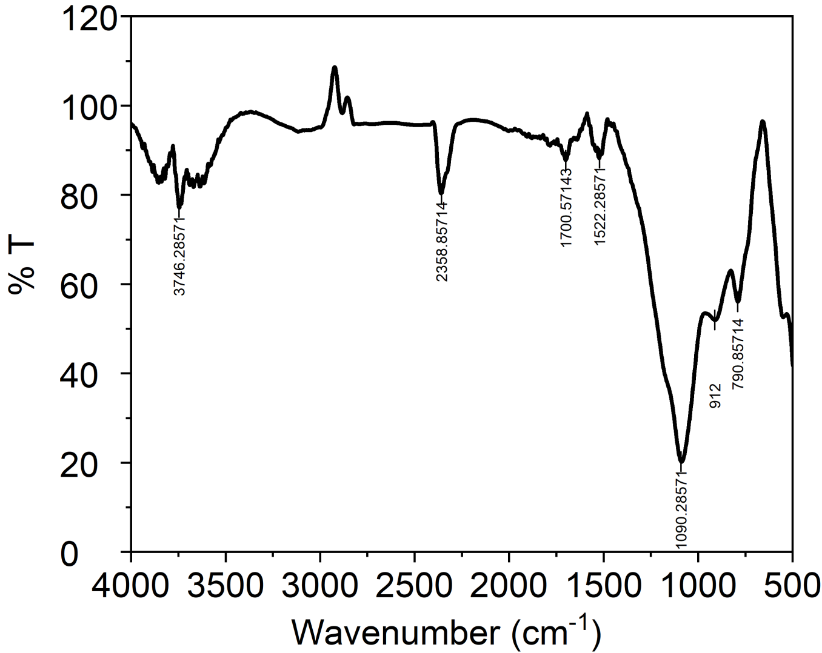


**Fig. S1:** FTIR spectra of SiNPs prepared from fly ash using hydrothermal treatment


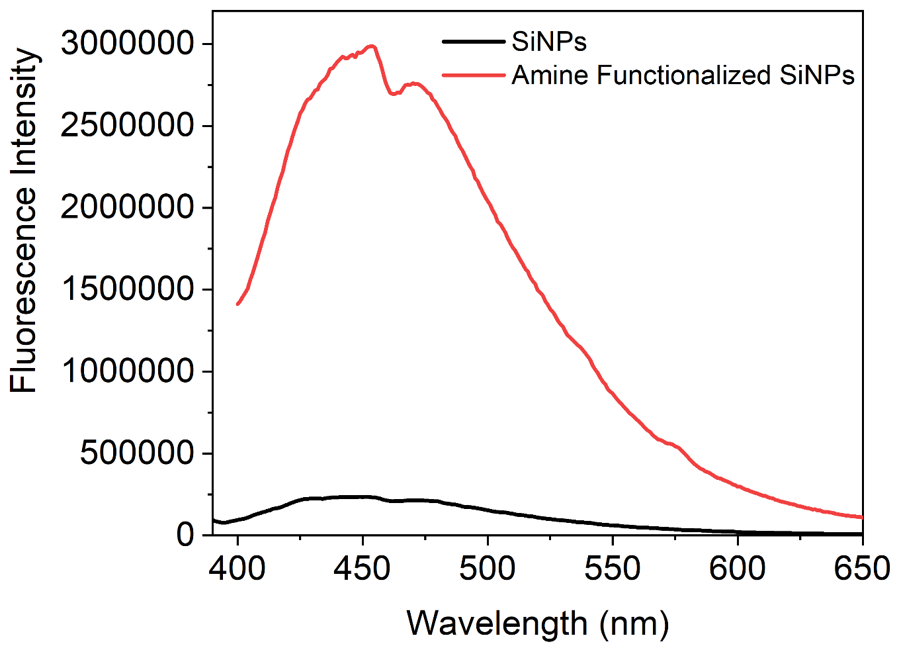


**Fig S2**: Fluorescence emission spectra of SiNPs without TEA (black line) and with TEA (red line).


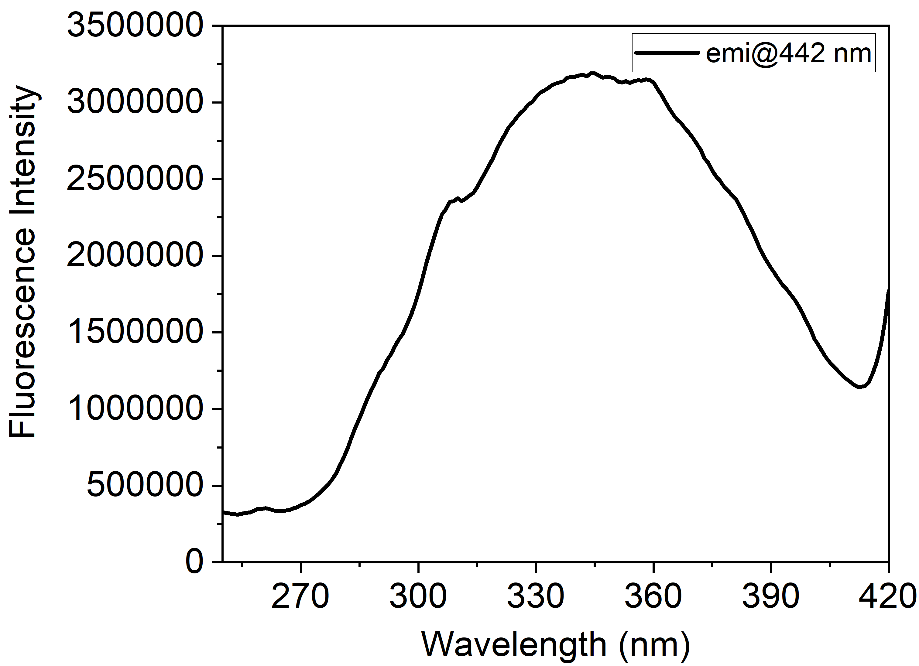


**Fig. S3:** Excitation spectrum of SiNPs in aqueous medium [SiNPs= 7.0 mg/mL].


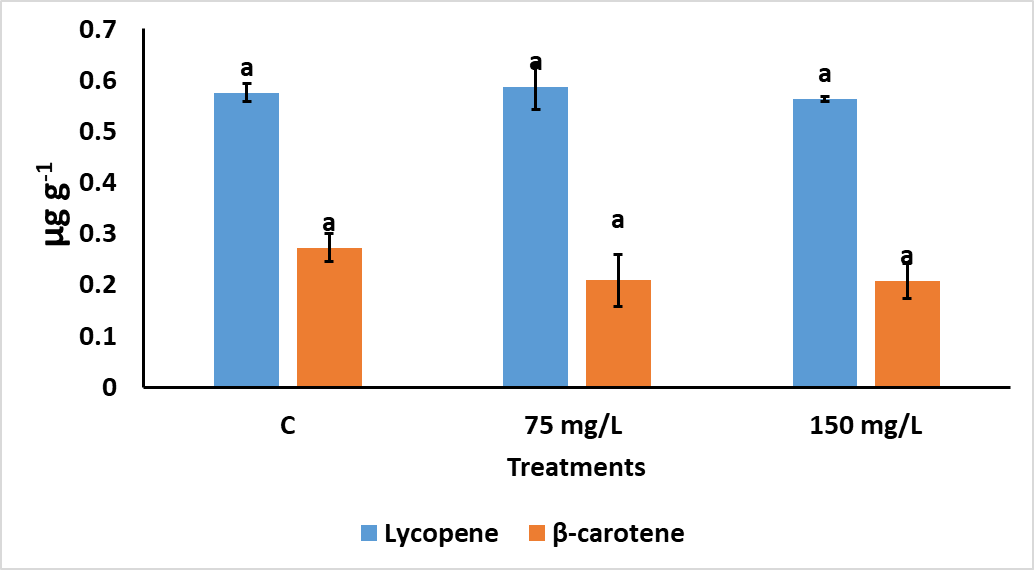


**Fig. S4:** Lycopene and β-carotene content in tomato fruit in the absence (control; C) and presence of SiNPs (75 mg/L and 150 mg/L).
